# Crimean-Congo Hemorrhagic Fever Virus, Northeastern Greece

**DOI:** 10.3201/eid1701.100073

**Published:** 2011-01

**Authors:** Anna Papa, Evangelia Tzala, Helena C. Maltezou

**Affiliations:** Author affiliations: Aristotle University of Thessaloniki, Thessaloniki, Greece (A. Papa);; Hellenic Centre for Diseases Control and Prevention, Athens, Greece (E. Tzala, H.C. Maltezou)

**Keywords:** Vector-borne infections, viruses, Nairovirus, Crimean-Congo hemorrhagic fever, Crimean-Congo hemorrhagic fever virus, Greece, seroprevalence, ticks, letter

**To the Editor:** Crimean-Congo hemorrhagic fever virus (CCHFV) causes a disease in humans that is characterized by fever and hemorrhagic manifestations, with death rates up to 30%. Humans are infected through tick bites or contact with the viremic blood of patients or livestock. CCHFV belongs to the genus Nairovirus (family *Bunyaviridae*), which contains 7 serogroups: CCHFV, Dugbe virus, Hughes virus, Sakhalin virus, Dera Ghazi Khan virus, Qalyub virus, and Thiafora virus.

A CCHFV strain, AP92, was isolated from *Rhipicephalus bursa* ticks collected in 1975 from goats in Vergina, a village in northern Greece (*1*). Seroprevalence among Vergina residents was 6.1% (*2*). During 1981–1988, the seroprevalence among 3,388 persons in Greece was 1.1% (range 0%–9.6%) (*3*). The first Crimean-Congo hemorrhagic fever case in Greece was reported in 2008, when a woman died in Komotini in northeastern Greece (*4*). The causative strain (Rodopi) differs from strain AP92 (*5*).

To determine the prevalence of CCHFV antibodies in the human population of northeastern Greece, serum samples were collected prospectively during November 2008–April 2009 from 1,178 residents of Drama, Kavala, Xanthi, Rodopi, and Evros prefectures. A predefined number of participants were enrolled in the study on the basis of prefecture population. Participants were selected randomly among persons who were referred to health care settings for blood testing, regardless of reason for testing, and regardless of CCHFV risk factors. Oral consent was given by all participants. A questionnaire was completed concerning age, sex, occupation, place of residence, history of tick bite, symptoms after the bite, contact with animals, and any other situation related with increased risk for tick bite. All age groups were included (range 0–97 years, mean ± SE age 53.2 ± 0.63).

Serum samples were tested for CCHFV immunoglobulin (Ig) G by ELISA (Vektor-Best, Koltsovo, Novosibirsk, Russia). The data were analyzed by using Stata Special Edition 9 (StataCorp LP, College Station, TX, USA). Multivariate logistic regression modeling was adopted to identify potential risk factors for acquisition of CCHFV infection. Odds ratios (ORs) with 95% confidence intervals (CIs) were obtained. p values <0.05 were considered significant.

In total, 37 (3.14%) of 1,178 persons were positive for CCHFV by IgG. The mean ± SE age of seropositive and seronegative persons was 68.7 ± 2.54 years (range 0–87 years) and 55.6 ± 0.79 years (range 0–97 years). The female:male ratio was 1.6 among tested persons and 0.6 among seropositive persons. Seroprevalence differed among prefectures: Rodopi, where the fatal Crimean-Congo hemorrhagic fever case was observed, and Evros had the highest values (4.95% and 4.49%), Drama and Xanthi the lowest (1.34% and 1.09%), and no IgG-positive person was found in Kavala. The distribution of regions where IgG-positive persons were found is presented in the Figure. Seropositive persons lived in rural areas at an altitude of 10m to 270 m; however, this factor was not significant (p = 0.358).

Crude analysis showed that age, sex, prefecture, occupation, contact with goats and sheep, slaughtering, and history of tick bite were significantly associated with seropositivity. Multivariate analysis showed that the following variables were significant risk factors for acquisition of CCHFV infection: age (OR 1.05, 95% CI 1.02–1.08; p = 0.002), residence in Rodopi prefecture (with Drama prefecture as reference category) (OR 6.55, 95% CI 1.36–31.60; p = 0.019), contact with goats (OR 3.40, 95% CI 1.22–9.43; p = 0.019), history of slaughtering (OR 2.53, 95% CI 1.01–6.45; p = 0.048), and history of tick bite (OR 2.51, 95% CI 1.03–6.15; p = 0.044).

When we compared our results with those of Antoniadis et al. (*3*), marked differences were seen: seroprevalence in Rodopi, Evros, Xanthi, and Drama was 0.5%, 0%, 1.2%, and 0%, respectively, compared with 4.95%, 4.49%, 1.09%, and 1.34% in the present study, which suggests that during the past 20 years CCHFV was introduced in some areas in Greece or increased its circulation in others. Climatic and environmental changes and infested livestock movements (legal or illegal) in a habitat suitable for ticks might play a role in the current situation (*6*).

Further studies are necessary to estimate the seroprevalence in the whole country and detect disease-endemic foci of the disease. In addition, surveys for CCHFV in Ixodid ticks are necessary to enable the construction of risk maps and risk assessment analysis.

**Figure:**
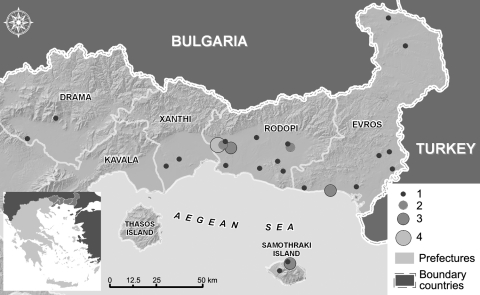
Five prefectures in northeastern Greece (inset), showing locations of persons who were immunoglobulin G–positive for Crimean-Congo hemorrhagic fever virus (solid circles), 2008–2009. Size of circle indicates number of persons with positive test results in each location.
